# Transferrin Receptor Controls AMPA Receptor Trafficking Efficiency and Synaptic Plasticity

**DOI:** 10.1038/srep21019

**Published:** 2016-02-16

**Authors:** Ke Liu, Run Lei, Qiong Li, Xin-Xin Wang, Qian Wu, Peng An, Jianchao Zhang, Minyan Zhu, Zhiheng Xu, Yang Hong, Fudi Wang, Ying Shen, Hongchang Li, Huashun Li

**Affiliations:** 1 West China Developmental & Stem Cell Institute, West China Second Hospital, State Key Laboratory of Biotherapy and Cancer Center, West China Hospital, Sichuan University, and Collaborative Innovation Center for Biotherapy, Chengdu, Sichuan 610041, China; 2 Shenzhen Key Laboratory for Molecular Biology of Neural Development, Laboratory of Developmental and Regenerative Biology, Institute of Biomedicine & Biotechnology, Shenzhen Institutes of Advanced Technology, Chinese Academy of Sciences, Shenzhen, Guangdong 518055, China; 3 SARITEX Center for Stem Cell Engineering Translational Medicine, Shanghai East Hospital, Tongji University School of Medicine & Advanced Institute of Translational Medicine, Shanghai 200123, China; 4 ATCG Corp., BioBay, Suzhou Industrial Park, Suzhou, Jiangsu 215123, China; 5 Department of Neurobiology, Key Laboratory of Medical Neurobiology of Ministry of Health of China, Zhejiang Province,Key Laboratory of Neurobiology, Zhejiang University School of Medicine, Hangzhou, Zhejiang 310058, China; 6 Department of Nutrition, School of Public Health, Zhejiang University, 866 Yuhangtang Road, Hangzhou 310058, China; 7 State Key Laboratory of Molecular Developmental Biology, Institute of Genetics and Developmental Biology, Chinese Academy of Sciences, Beijing 100101, China; 8 Department of Cell Biology & Physiology, University of Pittsburgh School of Medicine, Pittsburgh, PA 15261, USA

## Abstract

Transferrin receptor (TFR) is an important iron transporter regulating iron homeostasis and has long been used as a marker for clathrin mediated endocytosis. However, little is known about its additional function other than iron transport in the development of central nervous system (CNS). Here we demonstrate that TFR functions as a regulator to control AMPA receptor trafficking efficiency and synaptic plasticity. The conditional knockout (KO) of TFR in neural progenitor cells causes mice to develop progressive epileptic seizure, and dramatically reduces basal synaptic transmission and long-term potentiation (LTP). We further demonstrate that TFR KO remarkably reduces the binding efficiency of GluR2 to AP2 and subsequently decreases AMPA receptor endocytosis and recycling. Thus, our study reveals that TFR functions as a novel regulator to control AMPA trafficking efficiency and synaptic plasticity.

Transferrin receptor (TFR) is a trans-membrane glycoprotein for cellular iron uptake. It binds to diferric transferrin and transports iron-transferrin complex at cell surface followed by clathrin-mediated endocytosis. The molecular and cellular mechanisms of TFR-dependent iron uptake and iron homeostasis pathway have been extensively studied in erythrocyte, liver, intestine and immune systems^1^,^2^,^3^,^4^ and TFR has long been used as a clathrin mediated endocytic trafficking marker. However, the precise function of TFR in central nervous system (CNS) remains unclear.

Preliminary evidence implicates that TFR plays a role in CNS. TFR is expressed in several brain regions of mouse, enriched in endothelial cells^5^, choroid plexus cells and neuron. TFR seems to concentrate in certain neuronal pools such as cerebral cortex, pontine and motor neurons^6^. Particularly, TFR is polarized in neuronal dendrites but not axons^7^,^8^,^9^. Thus, selective enrichment of TFR in brain regions, neuronal subpopulations and subcellular domains suggest that TFR participates in different physiological processes.

Literature suggests that TFR plays a neuronal role. For example, TFR overlaps with synaptophysin before the establishment of neuronal cell polarity^10^. TFR-positive vesicles preferentially targeted to dendrites within matured neurons^11^. TFR along with SEP-GluR1 accumulates selectively on the spine plasma membrane following synaptic stimulation^12^,^13^. In addition, TFR-positive recycling endosomes translocate into dendrite spines following long-term potentiation (LTP) stimuli to supply AMPA receptor pools^14^,^15^. These studies indicate that TFR may be important for neuronal activity.

Here we demonstrate that conditional knockout of TFR in mouse neural progenitor cells leads to progressive epileptic seizure, accompanied by decreased synaptic transmission and LTP. We further find that loss of TFR increases neuronal surface expression of AMPA receptors and impairs AMPA receptor trafficking in neurons. In addition, loss of TFR decreases AP2-GluR2 interaction and co-localization. Together these results suggest that TFR recruits AMPA receptors via AP2 to control the efficiency of AMPA receptor trafficking and neural plasticity.

## Results

### TFR is Broadly Expressed in Mouse Brain and Enriched at Synapses

TFR is broadly expressed in CNS, but its precise spatiotemporal localization is not well characterized. We found that TFR expression was detected in mouse brain lysates as early as embryonic day 12.5 (E12.5), and reached the highest level between postnatal day 10 (P10) and P19 (Figure S1A). Notably, TFR along with other synaptic proteins including GluR1, GluR2, postsynaptic density protein 95 (PSD95), synaptophysin and α-Ca^2+^/calmodulin-dependent protein kinase II (CamKII) all reached the highest expression level within the time window (P10 to P19) of brain outgrowth and synaptogenesis (Figure S1A), indicating that TFR may be associated with early brain development. To determine the subcellular localization of TFR in neurons, we used high-speed centrifugation to isolate distinct subcellular components from whole brain lysates of P20 mouse. We found that subcellular distribution pattern of TFR was similar to other synaptic proteins (Figure S1B), which suggests that TFR may play a role in synaptic function.

**Figure 1:**
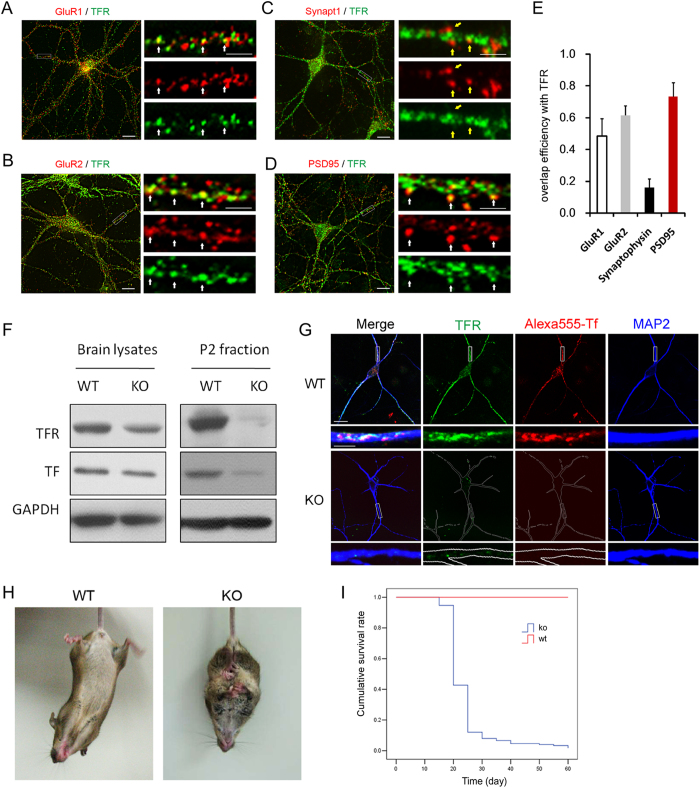
Conditional loss of TFR in mouse brain leads to epileptic seizure. (**A**) Partial co-localization of TFR with synaptic proteins at synapses in 14 *div* cultured hippocampal neurons. Co-immunofluorescence labeling of GluR1 (**A**) and GluR2 (**B**) with TFR indicates co-localization (yellow spots) at some synapses. Endogenous TFR was also co-stained with synaptophysin (synapt1) (**C**) or PSD95 (**D**) in 14 *div* cultured hippocampal neurons. White arrows indicate some co-localization sites. Yellow arrows indicate non-co-localization sites. Scale bar represents 20 μm in low-magnification panels and 5 μm in high-magnification panels. (**E**) Colocalization index of synaptic proteins (**A–D**) with TFR was quantified as overlap efficiency. (**F**) Ablation of TFR in mouse brain. At P14, both whole brain lysates and purified P2 fraction were detected with antibody against TFR and transferrin (TF) in western blotting. P2, crude synaptosomes. (**G**) TF uptake deficiency in TFR KO hippocampal neurons. 14 *div* live neurons were fed with Alexa-555 conjugated transferrin (200 μg/ml, Invitrogen) at 37 °C for 1 hr before stained for dendrite marker MAP2 (Blue) and TFR (Green). Upper panels, low magnification, scale bar = 20 μm; Lower panels, high magnification, scale bar = 5 μm. (**H**) TFR KO mouse displays abnormal hindlimb clasping reflex at P20. When picked up in tail, TFR KO mouse showed clasped hindlimb behavior rather than normal plantar reaction. (**I**) Survival curve of TFR KO mice. The earliest lethal seizure was observed at P13 in TFR KO mice. Total of 150 TFR KO mice were subject to Kaplan-Meier survival analysis.

Also immunofluorescence staining in hippocampal neurons cultured for 14 days (14 *div*) showed that TFR largely co-localized with the postsynaptic marker PSD95 (Fig. 1D) but rarely with pre-synaptic protein synaptophysin (Fig. 1C), and partially co-localized with AMPA receptor subunits GluR1 and GluR2 (Fig. 1E), the major excitatory glutamate receptors at synaptic sites (Fig. 1A,B). TFR and AMPA receptor co-localization was further confirmed by three dimensional microscopy reconstructions (Figure S1C,S1D). All these results indicate that TFR is enriched at neuronal postsynaptic sites and partially co-localizes with GluR1 and GluR2.

### Conditional Deletion of TFR in Mice Causes Neonatal Lethal Seizures

To determine the physiological function of TFR in CNS, we generated conditional TFR knockout (KO) mice (*TFR*^*flox/flox*^; Nestin-cre) by crossbreeding Nestin-cre mice with homozygous TFR floxed mice (*TFR*^*flox/flox*^)^16^. TFR deletion region was indicated by X-gal blue staining (Figure S2A,S2B). Nestin-cre mediated TFR KO led to a drastic decrease of TFR in P2 fractions but not in whole brain lysates (Fig. 1F). To exclude the interference of endothelial cells from whole brain lysates, we examined deletion efficiency in neurons. Compared to WT neurons, TFR KO neurons was negative for TFR staining and failed to absorb Alexa-555 conjugated transferrin (Fig. 1G), which demonstrated a high efficiency of TFR deletion.

TFR KO mice were viable at birth but showed body growth retardation at P18 (Figure S2C). 90% of the total (N = 150) TFR KO mice died from epileptic seizure between P13 and P25 (Fig. 1I), which was characterized by spontaneous muscle convulsion (Movie S1). Extended tongue and convulsed limbs were typical body appearance after a sudden death (Figure S2D). The rest of TFR KO animals died within two months after birth. Besides, abnormal hindlimb clasp was observed in all TFR KO animals at P20 (Fig. 1H and Movie S2).

Since TFR is crucial for iron uptake, we hypothesized that iron deficiency caused by TFR deletion can lead to epileptic phenotype. Then we investigated iron levels in P20 mice brain by enhanced Perl’s iron stain. Surprisingly, the intracellular iron level in TFR KO mice brain was similar to that in WT mice (Figure S3A). As for iron levels of cortical and hippocampal neurons (Figure S3C,S3D), we found no significant difference between WT and TFR KO animals (Figure S3B). Thus the epileptic phenotype in TFR KO mice might not be the result of iron deficiency, which indicates a more-than-iron role of TFR in CNS. Pathological investigations of TFR KO mice at P20 revealed no apparent morphological alterations of brain (Figure S4A,B). Meanwhile, antibodies against dendrite microtubule-associated protein 2 (MAP2) and pan-axonal neurofilament marker (SMI312) showed no obvious difference between WT and KO brain slices (Figure S4C).

Collectively, these results suggest that TFR is indispensable for early brain function and has more than iron-related roles in brain development.

### TFR deletion Reduces Synaptic Numbers

To investigate the non-iron roles of TFR in CNS, we first examined whether deletion of TFR could affect neuronal growth. We stained antibody against dendrite marker MAP2 on cultured 7 *div* and 12 *div* hippocampal neurons. Quantitative analysis of dendrite length showed no significant difference between WT and TFR KO neurons (Figure S5A). Then we transfected cultured neurons with pEGFP plasmid for 24 h to visualize axons. No obvious impairment of axonal growth was observed in TFR KO neurons (Figure S5B). Next we introduced Thy1-eGFP strain mice to both WT and TFR KO mice to observe synaptic morphology (Figure S5C), which is crucial for neuronal functions. Thy1-eGFP labeled hippocampus pyramidal neurons of P20 mice brain were imaged under high power objectives (63× oil). Morphological analysis showed that dendrite spine density was decreased in TFR KO mice while spine length was not affected (Figure S5D). Consistently, immunofluorescence staining in cultured hippocampal neurons with antibody against synaptic markers revealed that both synaptophysin and PSD95 puncta number was decreased in TFR KO mice (Figure S5E). Together these results suggest that TFR deletion does not impair the gross morphology of neurons but reduce dendrite spine numbers.

### TFR Deletion Increases Neuronal Surface AMPA Receptors

TFR has long been used as a marker for clathrin-dependent endocytosis^17^,^18^,^19^ , and as a marker co-trafficks with AMPA receptors in dendrite spines^20^. Since TFR co-localized with AMPA receptor GluR1, GluR2 and synaptic protein PSD95 (Fig. 1A,B,D), we wondered whether TFR was involved in synaptic protein trafficking.

**Figure 2:**
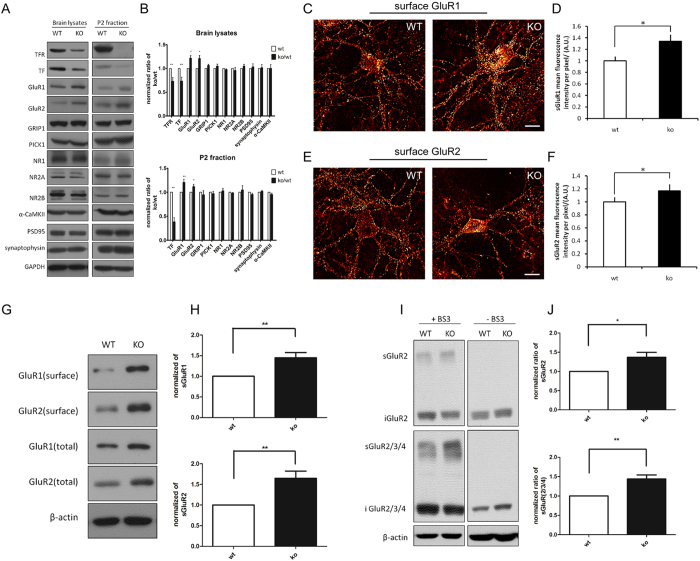
TFR deletion leads to increased surface AMPA receptor. (**A,B**) Western blots and quantification of synaptic proteins in whole brain lysates and P2 fractions from WT and TFR KO mice. The change of protein expression in KO is expressed as a KO to WT ratio. All WT values are normalized to one. Data represent mean ± SEM. Student’s t test, n ≥ 3, *p < 0.05, **p < 0.01, compared with WT mice. (**C–F**) Surface GluR1 (**C**) or GluR2 (**E**) in WT and KO 14 *div* cultured hippocampal neurons are shown as a glow scale from black (zero) to red (low pixel intensity) and white (high pixel intensity). Scale bar = 20 μm. Quantification of surface GluR1 (**D**) and GluR2 levels (**F**) (mean ± SEM) are shown in the right panel. Student’s t test, n = 10 neurons for each group, *p < 0.05, when compared with WT neurons. (**G**) Biotinylation of surface GluR1 and GluR2 in 14 *div* cultured hippocampal neurons from WT and KO mice. Surface GluR1 and GluR2 were biotinylated before extracted by NeutraAvidin beads. β-actin is used as loading control. At least three independent immunoblots are performed. (**H**) Quantification of surface GluR1 (n = 7) and GluR2 (n = 8) shown in (**G**) in WT and KO neurons (mean ± SEM, Student’s t test, **p < 0.01, when compared with WT neurons). (**I**) Surface and intracellular GluR2 pools of WT and KO hippocampus at P14 were assessed by using BS^3^-crosslinking methods. Antibody against GluR2 or GluR2/3/4 was used to confirm the results. Blank control experiments were processed without BS^3^. β-actin served as a loading control between WT and KO. Immunoblots were performed in three independent experiments. (**J**) Quantification of surface GluR2 (n = 6) and GluR2/3/4 (n = 9) shown in (**I**) in WT and KO brain tissues (mean ± SEM, Student’s t test, *p < 0.05, **p < 0.01, when compared with WT neurons).

First we analyzed different synaptic protein expression in whole brain lysates and P2 fractions from both P20 WT and KO mice. We found that AMPA receptor subunits GluR1 and GluR2 were both increased in whole brain lysates and in the P2 fraction of TFR KO mice brain versus WT littermates, accompanied by unchanged protein expression of GRIP, PICK1, α-CamKII, PSD95, and synaptophysin (Fig. 2A,B). The other excitatory glutamate receptor (NMDA receptor) expression level in TFR KO mice remained unchanged (Fig. 2A). Moreover, we found total and surface protein levels of NMDA receptor and GABA receptor were not changed in TFR KO mice (Figure S6). These results suggest that TFR might be more required for AMPA receptor trafficking.

Since AMPA receptor trafficking is highly dynamic at the neuronal surface, we then examined surface AMPA receptor levels in neurons. In cultured low-density TFR KO hippocampal neurons (n = 30), immunofluorescence staining with anti-N-terminal GluR1 or GluR2 antibodies revealed an increase in surface GluR1 (Fig. 2C,D) and GluR2 (Fig. 2E,F) compared to WT neurons. We also quantified surface AMPA receptor in both WT and KO 14 *div* neurons by surface protein biotinylation method. As a result, surface GluR1 and GluR2 were increased in TFR KO neurons (Fig. 2G,H). To confirm this result *in vivo*, we used antibodies against GluR2 or GluR2/3/4 to label surface and intracellular AMPA receptor pools in mice hippocampus lysates by Bis (sulphosuccinimidyl) substrate (BS^3^) crosslinking assay. The normalized surface GluR2 and surface GluR2/3/4 intensity was dramatically increased in TFR KO hippocampus compared with that in WT littermates (Fig. 2I,J). These results suggest that TFR deletion increases neuronal surface AMPA receptors.

### Loss of TFR Impairs Homeostatic Synaptic Scaling

**Figure 3:**
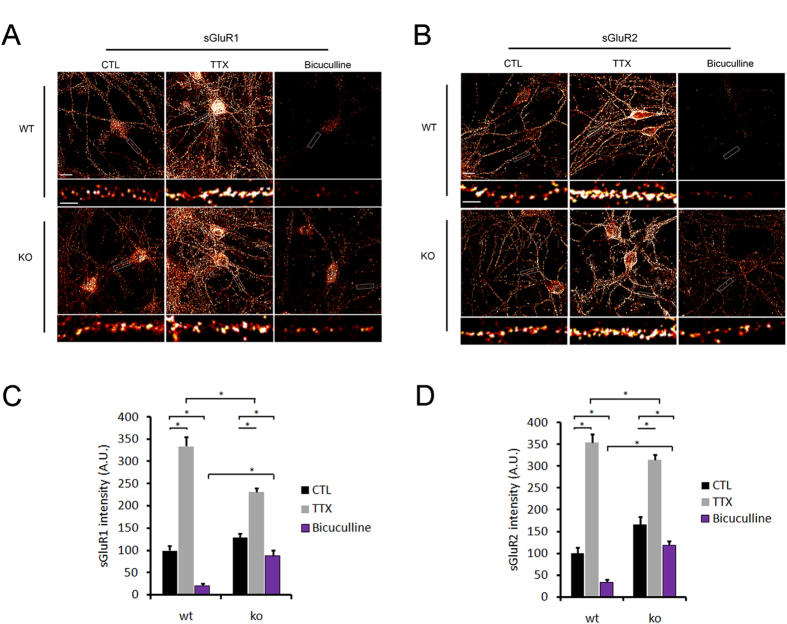
Homeostatic Synaptic Scaling is impaired in TFR KO Neurons. (**A,B**) Surface GluR1 (**A**) or GluR2 (**B**) in WT and KO 14 *div* cultured neurons treated with TTX (1 μM) or bicuculline (20 μM) for 48 hr. Normal growth medium was used as the negative control. Surface AMPA receptor expression is shown using a glow scale from black (zero) to red (low pixel intensity) and white (high pixel intensity). Low-magnification image scale bar = 20 μm. High-magnification image scale bar = 5 μm. Quantification analysis of surface levels of GluR1 (**C**) or GluR2 (**D**) in WT and KO neurons in (**A,B**) after TTX or bicuculline treatment (mean ± SEM). N = 10 cells for each group, *p < 0.05.

Homeostatic plasticity, or synaptic scaling, is the adaptive ability of neurons to regulate surface AMPA receptor numbers in response to external stimuli^21^,^22^,^23^. We wondered whether increased surface AMPA receptor may affect homeostatic synaptic scaling. To test this hypothesis, we used drug-induced inhibitory or excitatory stimuli for neurons to modulate synaptic scaling: TTX (1 μM, 48 hr) treatment (blocking all evoked neuronal activity) and bicuculline (20 μM, 48 hr) treatment (increasing neuronal firing rate by blocking GABA_A_-mediated inhibitory neurotransmission)^24^. TTX led to an increase in surface GluR1 (Fig. 3A) accumulation and bicuculline resulted in a lower surface levels of GluR1 in WT neurons (Fig. 3A,C). However, surface GluR1 in TFR KO neurons increase following TTX or reduction following bicuculline was greatly attenuated (Fig. 3A,C). We found a similar surface GluR2 (Fig. 3B) expression change in WT and TFR KO neurons (Fig. 3B,D) upon TTX or bicuculline treatment. These results suggest that loss of TFR impairs both surface AMPA receptors removal and re-insertion, which then perturbs homeostatic synaptic scaling.

### TFR is Required for Activity-dependent AMPA Receptor Internalization

**Figure 4:**
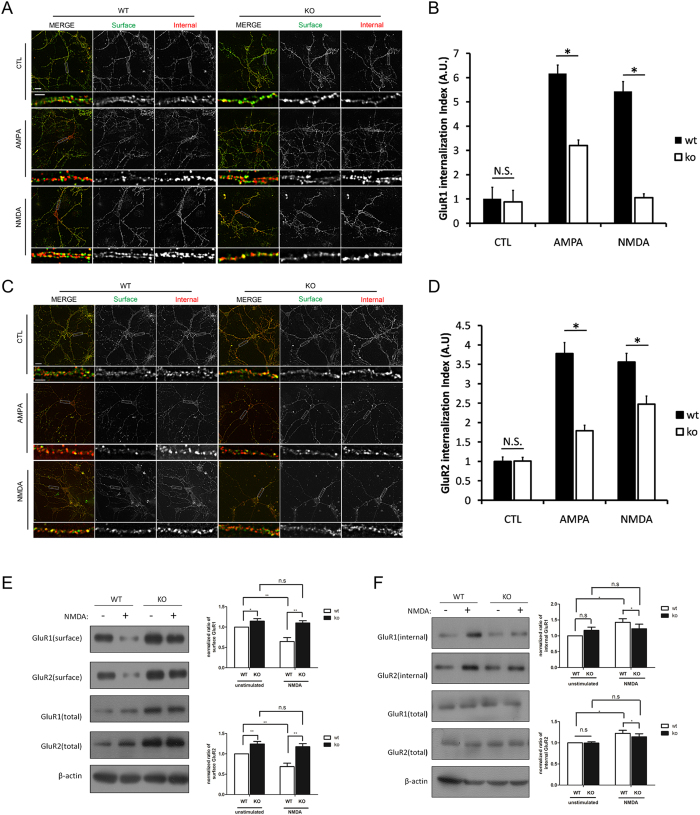
AMPA Receptor Internalization is reduced in TFR KO Neurons. (**A–D**) AMPA- and NMDA-induced internalization of AMPA receptor in WT or KO 14 *div* neurons. AMPA treatment (100 μM, 10 min) and NMDA treatment (50 μM, 10 min) induced robust internalization of GluR1 (**A**) or GluR2 (**C**) in WT neurons but less in KO neurons. Low-magnification image scale bar = 20 μm; High-magnification image scale bar = 5 μm. (**B,D**) Quantification of internalization index shown in (**A,C**). Internalization index is measured as the ratio of internalized fluorescence intensity to surface fluorescence intensity. Data represent mean ± SEM (n = 10 neurons for each condition, Student’s t test, *p < 0.05). (**E**) Surface AMPA receptor biotinylation in WT and KO neurons during NMDA stimulation. Surface GluR1 and GluR2 were biotinylated in 14 *div* neurons before NMDA stimulation (50 μM NMDA, 15 min). Normal growth medium was used as the negative control. Three independent experiments from WT and KO neurons were performed for both GluR1 and GluR2 (n = 5) (*p < 0.05; **p < 0.01; n.s, not significant). (**F**) Internalized AMPA receptor biotinylation in WT and KO neurons during NMDA stimulation. Surface GluR1 and GluR2 were biotinylated in 14 *div* neurons before NMDA stimulation (50 μM NMDA, 15 min). After a surface protein biotinylation washout, internalized GluR1 and GluR2 were extracted with NeutraAvidin for immunoblots. Three independent experiments from WT and KO neurons were performed for both GluR1 and GluR2 (n = 5) (*p < 0.05; n.s, not significant).

Surface AMPA receptor numbers are crucial for synaptic scaling. To test if surface AMPA receptor dynamics underlies the impairment of synaptic scaling in TFR KO neurons, we examined GluR1 and GluR2 internalization efficiency in steady-status and activity-dependent conditions. We performed an “antibody feeding” assay to quantify surface and internal pools of AMPA receptors in WT and KO neurons upon AMPA or NMDA stimulation. In brief, 14 *div* cultured hippocampal neurons were incubated with an anti-GluR1 (Fig. 4A) or anti-GluR2 (Fig. 4B) N-terminal antibody followed by treatment with AMPA (100 μM) or NMDA (50 μM) and CNQX (50 μM) for 10 min to trigger AMPA receptor endocytosis. Internalization index was measured as the internal to surface AMPA receptor ratio.

In non-stimulation cases, we observed no difference of GluR1 or GluR2 internalization index between WT and KO neurons (Fig. 4B,D). But when stimulated by either AMPA or NMDA, KO neurons displayed a robust decrease in GluR1 (Fig. 4B) and GluR2 (Fig. 4D) internalization index compared to WT neurons, suggesting regulatory endocytosis of AMPA receptor is reduced in TFR KO neurons. To further confirm these results, we examined surface receptor internalization efficiency by using protein biotinylation method. Neurons were first incubated with normal growth medium containing 50 μM NMDA for 15 min to induce AMPA receptors endocytosis. AMPA receptors remaining at the cell surface were biotinylated for calculation of internalization efficiency. We found that biotinylated surface GluR1 or GluR2 were significantly reduced upon NMDA stimulation in WT neurons but not in TFR KO neurons (Fig. 4E), which indicated a defect in the removal of surface AMPA receptor in TFR KO neurons.

We also measured internalization efficiency by quantifying internalized AMPA receptors. In brief surface AMPA receptors were biotinylated following stimulation by NMDA, then protein biotinylation at the cell surface were quenched by salt wash and the biotinylated receptors left inside cells were used for calculation. We found that internalized GluR1 and GluR2 were significantly increased upon NMDA stimulation in WT neurons but not in KO neurons (Fig. 4F). Together these results suggest that TFR is required for regulated AMPA receptor internalization.

**Figure 5:**
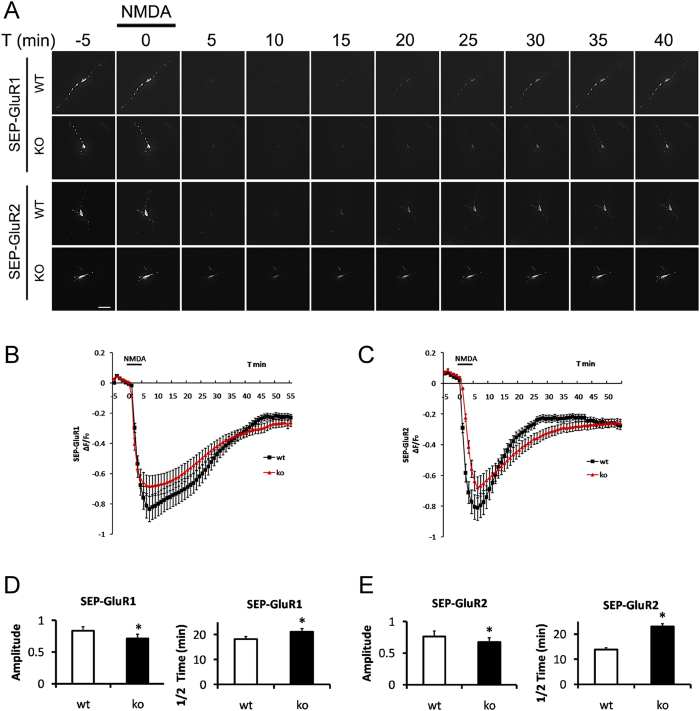
Live imaging of NMDA-Induced AMPA Receptor Internalization in TFR KO Neurons. (**A**) Time- lapse images of hippocampal neurons transfected with SEP-GluR1 or SEP-GluR2 and subjected to a NMDA (20 μM, 5 min)/washout cycle. Scale bar = 20 μm. Quantification of SEP-GluR1 (**B**) or SEP-GluR2 (**C**) fluorescence change in response to NMDA perfusion in **(A)** (mean ± SEM, n = 6 neurons for each experimental group from three individual experiments). Maximum amplitudes of SEP-GluR1 (**D**) or SEP-GluR2 (**E**) fluorescence intensity changes to NMDA stimulation and average recycling half-time (**D,E**) (T1/2, the time taken from maximum endocytosis to 50% recycling) after NMDA washout (mean ± SEM, n = 6 neurons from three experiments, *p < 0.05, compared with WT neurons, Student’s t test).

To directly visualize activity-dependent AMPA receptor internalization, we used pH-sensitive GFP variant super ecliptic pHluorin (SEP)^25^ fused at the N-terminal extracellular domain of GluR1 or GluR2 (SEP-GluR1, SEP-GluR2) as a fluorescent reporter in live imaging experiment. These reporters serve as a spatiotemporal indicator of AMPA receptor distribution by fluorescing at pH 7.4 at the plasma membrane and becoming non-fluorescent in intracellular endosomes where the pH is less than 6.0^26^. It allows us to image entire pool of AMPA receptor in the steady-status and to selectively quantify receptor internalization at the plasma membrane in response to NMDA stimulation^12^,^27^. Both WT and KO neurons can internalize surface SEP-GluR1 and SEP-GluR2 upon NMDA stimulation and recycle receptors back to cell surface during washout perfusion (Fig. 5A and Movie S3). We observed decreased maximum internalization amplitude and increased half recycling time of SEP-GluR1 (Fig. 5B,D) or SEP-GluR2 (Fig. 5C,E) in TFR KO neurons versus WT neurons. These results suggest that TFR is required for both AMPA receptor internalization and membrane re-insertion.

Together all these data above support that TFR is important for activity-dependent AMPA receptors internalization.

### Loss of TFR Decreases AP2-GluR2 Interaction

AMPA receptors are very dynamic at neuronal surface and both recycling and internalization contribute to the net balance of surface AMPA receptors^28^. Our results suggest that TFR KO leads to decreased AMPA receptor recycling ability, suggesting TFR as a positive regulator of AMPA receptor trafficking. Thus, surface AMPA receptor accumulation in TFR KO neurons might be explained by that TFR deletion impairs AMPA receptor internalization process, which prevents AMPA receptor removal from neuronal membrane.

**Figure 6:**
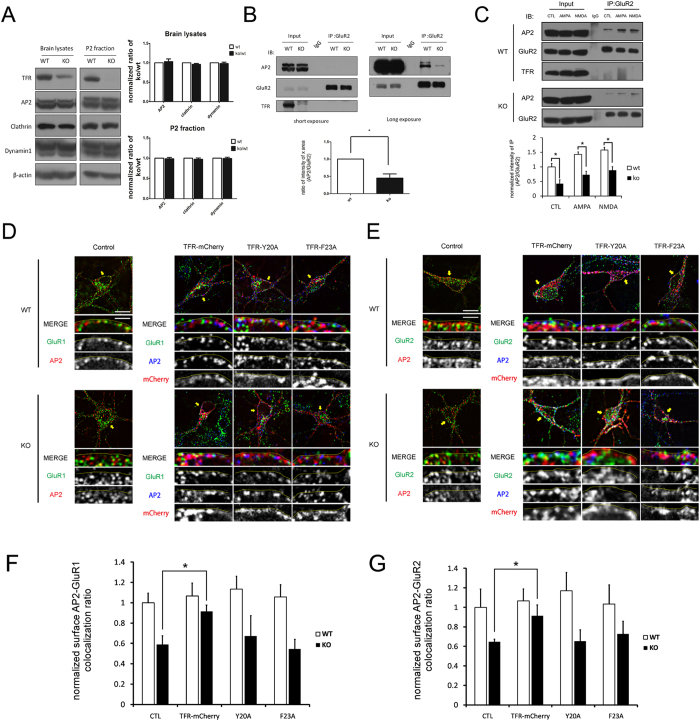
TFR regulates AP2-GluR2 binding efficiency via AP2 recruitment. (**A**) Total expression level of endocytic machinery proteins in TFR KO neurons. AP2, clathrin and dynamin were imunoblotted in mice whole brain lysates and P2 fractions. Protein level changes in TFR KO neurons is measured as a ratio of KO to WT (n = 3). (**B**) AP2-GluR2 interaction is reduced in P2 fractions of P14 TFR KO mice brain. Antibody against GluR2 C-terminus was used in the pull-downs and then imunoblotted with AP2 antibody. (Three experiments from three pairs of WT and KO mice, Student’s t test, *p < 0.05). (**C**) Co-immunoprecipitation of GluR2 with AP2 (antibody against AP2 μ2 subunit) in normal and stimulated 14 *div* cultured neurons from WT and TFR KO mice. Endogenous GluR2 was immunoprecipitated with the anti-GluR2 antibody, and the co-immunoprecipitated AP2-subunits were detected by AP2 antibody in control (normal growth medium), AMPA- (100 μM, 10 min) and NMDA- (50 μM, 10 min) treated neurons. TFR-GluR2 interaction signal cannot be detected by immunoblotting with TFR antibody (Invitrogen). (**D,E**) AP2 particle distribution at cell surface of 14 *div* neurons transfected with different TFR constructs. 14 *div* primary neurons were transfected with TFR-mCherry, TFR-Y20A or TFR-F23A constructs for 24 hr (non-treatment as control group) and then fixed for immunofluorescence staining with AP2, GluR1 or GluR2. Yellow arrows indicate where high magnification images taken from. Dot lines represent the cell surface, below which is the intracellular domain. Scale bar represents 20 μm in low magnification panel and 5 μm in high magnification panel. (**F,G**) AP2 co-localization ratio with GluR1 and GluR2. Co-localization ratio is measured as percentage of AP2-positive GluR1 or GluR2 particles and normalized non-transfection WT neurons as the control group. *P < 0.05.

Many endocytic machinery proteins and PDZ-containing proteins control AMPA receptor internalization^29^,^30^,^31^,^32^,^33^,^34^. To test whether TFR directly binds to AMPA receptor subunits, we used GluR2 antibody to immunoprecipitate TFR proteins in brain fractions. The immunoprecipitation did not detect the binding of GluR2 to TFR (Fig. 6B), suggesting that TFR does not directly interact with AMPA receptors.

Clathrin-mediated endocytosis (CME) is an essential process for AMPA receptor internalization at synapses^35^,^36^,^37^. It requires the formation of clathrin-coated pits (CCPs) and recruitment of endocytic machinery proteins such as clathrin and adaptin-2 (AP2). Previous literature suggest that local clustering of TFR at cell surface promotes CCPs initiation^38^, AP2-μ2 subunit directly interacts with the C-terminus of GluR2 during endocytosis^39^ and TFR directly associates to AP2^40^. Thus, we hypothesize that TFR might control AMPA receptor endocytosis via facilitating AP2-dependent recruitment of AMPA receptors to CCPs^41^.We examined endocytic proteins from whole brain lysates and P2 fractions in WT and KO mice. AP2, clathrin and dynamin did not show changes between WT and KO animals (Fig. 6A). Moreover, surface AP2 levels in brain slice and neurons were not changed in TFR KO mice (Figure S6). It suggests that TFR deletion does not impair major endocytosis machinery proteins.

GluR2-AP2 interactions could be detected by AP2 co-immunoprecipitation with GluR2 (Figure S7) and was confirmed by GluR2 co-immunoprecipitation with AP2 (Fig. 6B). Remarkably, we found that GluR2/AP2 binding strength was greatly reduced in TFR KO mice compared with WT ones (Fig. 6B,C). Furthermore, we found a stronger GluR2-AP2 interaction upon AMPA and NMDA stimulation in WT mice. Particularly, this stimulus-enhanced GluR2-AP2 binding was also observed in TFR KO mice but was much less than WT ones (Fig. 6C). It suggests that TFR plays a positive role in GluR2-AP2 binding during both steady-status and regulatory receptor trafficking.

Next we hypothesized that TFR could regulate membrane AP2 recruitment to CCPs in AMPA receptor internalization. Immunofluorescence staining (Fig. 6D,E) showed that GluR1/AP2 and GluR2/AP2 co-localization ratio at the surface of TFR KO neurons were less than that in WT neurons, which could partly be rescued by TFR-mCherry transfection but not by previously characterized AP2 binding defective mutant TFR-Y20A and TFR-F23A constructs (Fig. 6F,G)^42^. These results taken together indicate that TFR-AP2 interaction is crucial for efficient GluR2-AP2 binding and possibly subsequent GluR2/AP2 recruitment in CCPs.

### Loss of TFR Reduces Basal Synaptic Transmission

**Figure 7:**
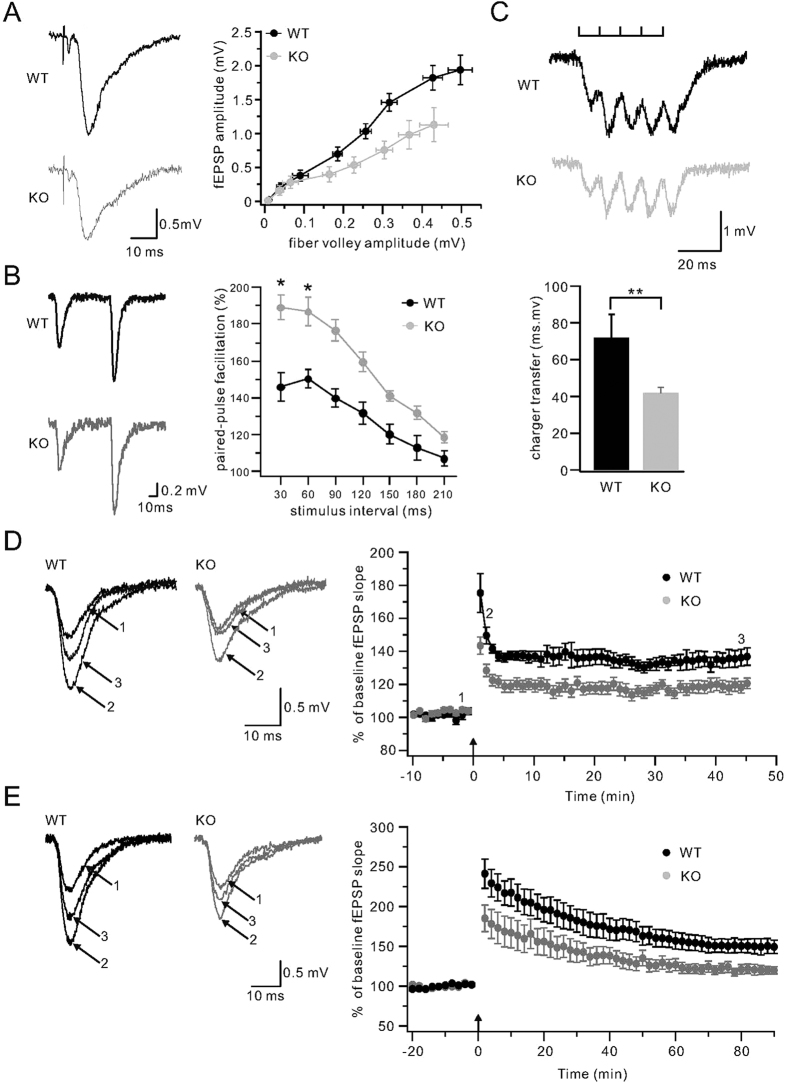
Basal synaptic transmission and LTP are perturbed in TFR KO mice. (**A**)Analysis of synaptic input/output (I/O) relation between the fiber volley amplitude and fEPSP amplitude, which was determined over a narrow range of stimulus intensities. Each point represents the mean of all slices tested (WT: n = 15; KO: n = 12). Representative recordings are displayed in the left, marked as WT (black) and KO (grey). Note that amplitudes of both FV and fEPSP decreased. In the right, each point of X-Y pair represents the response derived from the same stimulus intensity. (**B**) The comparison of paired-pulse facilitation (PPF) in WT and TFR KO mice. Two representative traces derived from WT (black) and TFR KO (KO, grey) stimulated by paired pulses with an interval of 60 ms are shown in the left. Note that the ration of fEPSP2/fEPSP1 is bigger in KO compared to WT. The right panel shows the statistics of PPF obtained at different stimulus intervals (30, 60, 90, 120,150, 180 and 210 ms) in WT (black) and KO (grey) groups. Note that PPF is generally bigger in KO group at all stimulus intervals. (**C**) Analysis of train stimulation waveforms reveal a smaller increase of fEPSP slope in TFR KO mice compared with WT mice. Representative traces in response to train stimuli in WT (black) and KO (grey) mice are shown in the upper panel. Analysis of charge transfer of 5 spikes is shown in the histogram below. (**D**) Field potential recordings were made in stratum radiatum to measure LTP induced by 100 Hz stimuli (Tet; 1 s) at t = 0 min. Sample traces from representative cells are from the numbered points indicated. Each trace is the average of three consecutive responses. (**E**) An LTP experiment similar to that shown in (**D**), except that the conditioning stimulation delivered at t = 0 min consisted of a much stronger tetanus, theta burst stimuli (TBS). Sample traces from representative cells are from the numbered points indicated. Each trace is the average of three consecutive responses.

The proper control of AMPA receptor surface removal and membrane recycling is crucial for synaptic transmission^20^,^43^,^44^,^45^,^46^. We wondered whether TFR is required for synaptic transmission and plasticity. Basal excitatory synaptic transmission was examined in WT and TFR KO hippocampal slices by measuring field excitatory postsynaptic potentials (fEPSPs), which was elicited at a rate of 0.05 Hz by stimulation of Schaffer collateral/commissural fibers in the CA1 region. We first compared the amplitudes of fEPSP and fiber volley (FV) in WT and KO slices. The amplitude of FV is an indicator of axonal excitability^47^. We find that both fEPSP and FV were reduced in KO mice under the same stimulation intensity, as shown by the representative traces in Fig. 7A. By performing the stimuli at a series of increasing intensities, we established an input-output (I/O) relation between FV and fEPSP slope (Fig. 7A), where each point represents the mean of all slices tested. Our results showed that both fEPSP and FV were reduced at all stimulus intensities tested (Fig. 7A). This result suggests that both presynaptic transmitter release and postsynaptic synaptic response are reduced in TFR KO mice.

To confirm the presynaptic defect caused by TFR deletion, we examined paired pulse facilitation (PPF), the enhancement of a second EPSP relative to the first, elicited by two successive stimulation pulses. The facilitation reflects an enhancement of transmitter release from presynaptic terminals^48^,^49^. In WT slices (n = 17) maximal PPF occurred when the interpulse interval was 60 ms (Fig. 7B), and the facilitation declined when the interval between two pulses continued to increase (Fig. 7B). In TFR KO slices (n = 14) there was a significant increase in PPF, particularly at shorter intervals (30, 60, and 90 ms, Fig. 7B). These results also indicate that the presynaptic release is reduced in TFR KO mice.

We also examined the short-term plasticity in TFR KO mice compared with WT mice by delivering train stimuli of 5 bursts at 100 Hz during field potential recording. Amplitudes of fEPSPs evoked by stimulation in the CA1 region were constant over extended periods of low frequency (0.1 Hz) stimulation. 100 Hz stimulation produced potentiated fEPSPs (Fig. 7C) in WT slices. Although this short-term potentiation was most pronounced at the last spike, the peak amplitude of spikes varied in different slices. Hence, the total charge transfer of 5 spikes was calculated to quantify this process. We found that the total charge transfer was decreased in TFR KO mice (n = 25) compared to the WT mice (n = 25). These data also support that the presynaptic transmitter release was reduced.

### Loss of TFR Reduces LTP at CA1 Synapses

We next used field potential recordings to measure long-term potentiation (LTP) in CA1 region. fEPSPs were stimulated by test pulses delivered at 0.05 Hz. After recording stable baseline responses for 10 min, we delivered high-frequency stimulation (100 Hz, 1 s) and then test pulses were resumed for an additional 50 min (Fig. 7D). In this situation, LTP evoked in WT synapses was significantly larger than that evoked in TFR KO synapses (Fig. 7D). This difference was apparent soon after titanic stimulation and continued throughout the (Fig. 7D). This experiment indicated that TFR is important for the high-frequency-induced CA1 LTP.

High frequency stimulation as used here is a weak form of synaptic activation which gives rise to a small form of LTP. To determine if TFR KO synapses would also show impaired LTP in response to a robust stimulation, we repeated LTP experiments using theta-burst stimuli. Recordings from WT synapses revealed robust LTP with a strong initial phase and a sustained late phase (Fig. 7E). In contrast, TFR KO synapses show a significant reduction of both the early and late phases of LTP (Fig. 7E). In summary of LTP data obtained from 100 Hz and theta-burst stimuli, we conclude that the TFR KO impairs the LTP induction at hippocampal CA1 synapses. Based on the electrophysiological data presented above, we conclude that TFR is crucial for both presynaptic and postsynaptic transmission.

## Discussion

TFR is an important iron transporter. Surprisingly, iron level in neurons of TFR KO mice remains almost the same as that in WT animals (Figure S3). Considering that western blot and immunofluorescence results (Fig. 1F,G) show a complete deletion of TFR in neurons, it raises an interesting question that why there are still so much iron left. The fact that TFR KO neurons can still absorb irons indicates that other iron transport pathway remains intact in CNS, which could be supported by other researches. First, large numbers of endothelial cells in CNS are not targeted by Nestin-cre driven TFR knockout. These endothelial cells formed brain blood barrier (BBB) and are highly expressed with TFR. In TFR KO mice, trans-BBB iron flow (from peripheral blood to CNS) still exists as the largest iron source^50^. Second, neurons without TFR can still absorb irons by other iron carriers and transporters such as DMT1(divalent metal transporter 1)^51^, LfR(lactotransferrin receptor)^52^, MTF (melanotransferrin)^53^ and CP (ceruloplasmin)^54^. Third, oligodendrocytes, astrocytes and microglial cells all have supportive roles in iron transport^55^,^56^,^57^. Therefore, conditional knockout of TFR might not strongly impair iron homeostasis in CNS. It turns out to provide a precious opportunity for study on TFR’s non-iron-transport role in CNS.

In fact, we found that specific deletion of TFR in neural progenitors leads to lethal seizures (Fig. 1H,I and Movie S1) that occurs to 3 weeks old KO animals. Further investigation showed that gross brain morphology is not apparently perturbed (Figure S3) but excitatory glutamate receptor-AMPAR is surprisingly increased (Figure 2). Clearly, TFR is indispensable for brain function at early development stage. And in this case, there could be a rational link between epileptic seizure and increase of AMPA receptors. Epileptic seizure mainly occurs as abnormal synchronous discharges of neuronal networks. We only found pathological changes of AMPA receptors but not other synaptic receptors (NMDAR or GABAR) in TFR KO mice. And AMPA receptors are major excitatory glutamate receptors mediating fast excitatory synaptic transmission in CNS. Thus it is very likely that increase of AMPA receptor can lead to abnormal neuron firing in TFR KO mice. And there are many basic and clinical researches supporting the idea that AMPA receptor is a molecular target in epilepsy therapy^58^. Moreover, commercial drug perampanel as the noncompetitive AMPA receptor antagonist is efficacious in the treatment of human seizures^59^. Therefore, lethal seizures of TFR KO mice might be the result of abnormal AMPA receptor increase.

The molecular mechanism underlying regulation of TFR on AMPA receptors remains elusive when we detected no interaction (Fig. 6B,C) between TFR and GluR2, a general AMPA receptor subunit. But we found that loss of TFR reduces GluR2-AP2 binding strength and colocalization, which can be rescued by wild-type TFR but not the AP2 binding defective TFR mutant. Thus our study provides the first evidence that TFR functions as a regulator for AMPA receptor trafficking. However, it is not very clear that how exactly TFR regulate the removal of AMPA receptors from the cell surface. Previous studies have reported that both TFR^40^ and GluR2^39^ can interact with AP2 during endocytosis. Our study shows that deletion of TFR will significantly reduce GluR2-AP2 colocalization and impairs GluR2-AP2 binding strength. Thus, it might indicate that TFR, GluR2 and AP2 have some special relations during AMPA receptor trafficking. As for AP2 is a general adaptor protein enriched in all cells, TFR could not have competitive relation with GluR2 for AP2. An alternative hypothesis is that that TFR indirectly regulates GluR2-AP2 binding through other factors. There is one clue that a calcium sensor -hippocalcin can co-immunoprecipitate with GluR2, TFR and AP2 in the presence of calcium^41^. Particularly, TFR forms dimers and GluR2 forms heterotetramers with other AMPARs in physiological conditions. Thus, it might be possible that a large protein complex might exist in some way including calcium, irons or other ions.

Also electrophysiological data show that both fEPSP and FV were reduced in TFR KO brain slice (Fig. 7A), suggesting that both presynaptic transmitter release and postsynaptic synaptic response are impaired in TFR KO mice. The presynaptic defect was further confirmed by PPF assay and the short-term plasticity assay (Fig. 7C) as well as LTP upon low-and high-frequency stimulation in TFR KO mice (Fig. 7D). Thus it seems confusing that TFR KO reduces synaptic transmission while increases postsynaptic AMPA receptors levels. However, synaptic density of hippocampal neurons is reduced in TFR KO mice (Figure S5C,S5D). Presynaptic protein synaptophysin and postsynaptic protein PSD95 are also decreased in culture TFR KO neurons (Figure S5E). Thus consistently, less synapses in TFR KO mice can result in reduced synaptic transmission. Meanwhile, increased AMPA receptors might not enter reduced synaptic sites; instead they might locate at dendrite shaft and neuronal soma membranes and become silent during stimulations. It indicates that TFR KO neurons have increased cell surface AMPA receptors but have less active synaptic sites and less active AMPA receptors. Therefore reduced postsynaptic sites of one neuron will reciprocally lead to reduced presynapses activity of other neurons by negative feedback loop or changes of synaptic plasticity. Thus presynaptic transmitter release can be affected even if TFR is present at postsynaptic sites.

In conclusion, our study shows that TFR functions as a novel regulator to affect AMPA receptor trafficking via modulating GluR2-AP2 interaction and consequently control synaptic transmission and plasticity.

## Materials and Methods

### Animals

Generation of conditional TFR KO mice was obtained by crossing *TFR flox/flox* homozygous mice (from Nancy C. Andrews)^16^ with *Nestin-cre* mice (Jackson Lab, strain code_003771) . Mice were maintained under standard SPF grade and all procedures were performed in accordance with the West China Second Hospital of Sichuan University, Development and Stem Cell Research Institute Guide for the Care and Use of Laboratory Animals and under the approval of the West China Second Hospital Animal Care and Use Committee. See the **Extended Experimental Procedures** for primers used in genotyping.

### Primary Neuronal Cell Cultures

Hippocampi from E14.5-E18.5 mice embryos were dissected and dissociated with 0.25% trypsin for 15 min at 37 °C, and then seeded onto the coated coverslips (Warner Instrument, overnight coating with modified coating solution: 17 μM acetic acid, 100 μg/ml poly-D-lysine and 60 μg/ml rat tail collagen in 0.01M PBS) at 10,000 per well in maintenance medium (Neurobasal medium with B27 and 1% GlutaMax) which were half replaced with fresh medium every 4 days.

### NMDA-induced AMPA receptor endocytosis

To quantify the surface receptor after NMDA stimulation, high density neurons were treated with 1 μM TTX for 1 hr at 37 °C and washed with artificial cerebro-spinal fluid (ACSF), and then treated with 100 μM NMDA at 37 °C for 15 min to induce surface receptor endocytosis. The remaining surface receptors were then biotinylated for 30 min at 4 °C and analyzed by immunoblots as previously described^31^,^60^. For measuring internalized surface receptor upon NMDA stimulation, high density neurons were treated with 1 μM TTX for 1 hr at 37 °C and washed with ACSF and then cooled down to 4 °C. Neurons were biotinylated for 30 min at 4 °C, washed with ACSF, and treated with 100 μM NMDA in growth medium at 37 °C for 15 min to induce surface receptor endocytosis. Biotin remaining at the cell surface was cleaved by incubating with MESNA (Sodium 2-mercaptoethane-sulfonate) stripping buffer (50 mM MESNA, 150 mM NaCl, 1 mM EDTA, 0.2% BSA, and 20 mM Tris, pH 8.6) at 4 °C for 30 min and washed with PBS, and then analyzed as previously described^31^,^60^.

### Live Imaging

To visualize surface AMPAR internalization in live neurons, we transfected neurons with SEP-GluR1 or SEP-GluR2 and measured maximum internalization amplitude and 1/2 recycling time as described previously^26^.

### Statistical Analysis

At least three experiments are used for quantification. SPSS 15.0 software was used for statistical analysis, and all data passed normality tests. All Student’s t tests were performed assuming Gaussian distribution and p < 0.05 is considered to be statistically significant.

## Additional Information

**How to cite this article**: Liu, K. *et al*. Transferrin Receptor Controls AMPA Receptor Trafficking Efficiency and Synaptic Plasticity. *Sci. Rep.* **6**, 21019; doi: 10.1038/srep21019 (2016).

## Supplementary Material

Supplementary Information

Supplementary Movie S1

Supplementary Movie S2

Supplementary Movie S3
