# Supplementary material for: Transferrin Receptor Controls AMPA Receptor Trafficking Efficiency and Synaptic Plasticity
Source: Sci Rep. 2016 Feb 16;6:21019. doi: 10.1038/srep21019 (PMC4754636; doi:10.1038/srep21019)

Supplementary Information

Title:

Transferrin Receptor Controls AMPA Receptor Trafficking Efficiency and Synaptic Plasticity

Author List:

Ke Liu[1,2,3,4,9], Run Lei[1,2,3,4,9], Qiong Li[5], Xin-Xin Wang[5], Qian Wu[6], Peng An[6], Jianchao Zhang[2], Minyan Zhu[3,4], Zhiheng Xu[7], Yang Hong[8], Fudi Wang[6*], Ying Shen[5*], Hongchang Li[1,2,*] & Huashun Li[1,2,3,4*]

[1] West China Developmental & Stem Cell Institute, West China Second Hospital, State Key Laboratory of Biotherapy and Cancer Center, West China Hospital, Sichuan University, and Collaborative Innovation Center for Biotherapy, Chengdu, Sichuan 610041, China.

[2] Shenzhen Key Laboratory for Molecular Biology of Neural Development, Laboratory of Developmental and Regenerative biology, Institute of Biomedicine & Biotechnology, Shenzhen Institutes of Advanced Technology, Chinese Academy of Sciences, Shenzhen, Guangdong 518055, China.

[3] Tongji University School of Medicine & Advanced Institute of Translational Medicine, Shanghai 200123, China

[4]ATCG Corp., BioBay, Suzhou Industrial Park, Suzhou, Jiangsu 215123, China.

[5]Department of Neurobiology, Key Laboratory of Medical Neurobiology of Ministry of Health of China, Zhejiang Province,Key Laboratory of N eurobiology, Zhejiang University School of Medicine, Hangzhou, Zhejiang 310058, China

[6]Department of Nutrition, School of Public Health, Zhejiang University, 866 Yuhangtang Road, Hangzhou 310058, China

[7] State Key Laboratory of Molecular Developmental Biology, Institute of Genetics and Developmental Biology, Chinese Academy of Sciences, Beijing100101, China

[8] Department of Cell Biology & Physiology, University of Pittsburgh School of Medicine, Pittsburgh, PA 15261, USA.

[9] Those authors contributed equally to this work.

Corresponding address: Tongji University School of Medicine, 1239 Siping Road, Shanghai 200123, China.

*Correspondence and requests for materials should be addressed to Hongchang Li (hc.li@siat.ac.cn) or Huashun Li (huashunli@tongji.edu.cn)

Figure S1.

Distribution of TFR in brain tissue and neurons, Related to Figure 1

(A) Synaptic protein expression in various stages of mouse brain development. Dot line rectangle represents time window of early synaptogenesis. E, embryonic day; P, postnatal day. Red arrows indicate non-specific bands. **(B)** TFR distribution in subcellular fractions of P20 mouse brain. After fractionation, TFR, GluR1, GluR2, α-CamKII, post-synaptic (PSD95) and presynaptic (synaptophysin) markers were detected by immunoblotting. S1, supernatant of the homogenate at low-speed centrifugation; P1, nuclei and large debris of the corresponding pellet from S1; S2 supernatant of S1 subjected to medium-speed centrifugation; P2, crude synaptosomes of the corresponding pellet from S2; S3, cytosol, which corresponds to the supernatant of S2 subjected to high-speed centrifugation; P3, light membranes, corresponding pellet of S3; LP1, synaptosomal membranes; LS2, synaptic cytosol; LP2, synaptic vesicle-enriched fraction. **(C and D)** Three dimensional co-localization of TFR with GluR1 and GluR2.TFR-mCherry colocalized with GluR1 **(C)** or GluR2 **(D)** at dendrite endosomes. Cultured 14*div*primary neurons were transfected with TFR-mCherry and stained with antibodies against anti-GluR1 and anti-GluR2. White circle represents intersected region of three orthogonal planes (XY, YZ and XZ).

Figure S2

Supplemental data of TFR KO mice, Related to Figure 1

(A) Nestin-cre expression efficiency and pattern is shown by X-gal staining for LacZ reporter gene in transgenic mouse. a, cortex; b hippocampus; c, thalamus; d, hypothalamus. (10X objective) **(B)** Nestin-cre expression pattern is revealed by LacZ signal in the whole brain slice. **(C)** Postnatal body weight gain of TFR WT and KO mice. TFR KO mice exhibit a significant and progressive reduction in body weight compared to WT mice (n = 10) at P18 (*P < 0.05 by Student's t-test). **(D)** After a sudden death, TFR KO mice have typical symptom of severe convulsion from epileptic seizure. i, body appearance after death caused by intensive seizure; ii, extended tongue; iii and iv, stiff and crouched paws. Scale bar, 1 cm.

Figure S3.

Enhanced Perl's stain for neuronal iron level on P20 WT and TFR KO mice brain, Related to Figure 1

Paraffin sections of P20 mice brain were subjected to Perl's stain and enhanced by 3,3'-diaminobenzidine (DAB). Sections were imaged under different magnification microscopy including 4X **(A)**, 10X, 20X and 40X **(C and D)** objectives. **(B)** Iron levels of neurons under 40X objective from cortex and hippocampus was quantified and analyzed (n=100 neurons for each group, mean ± SEM, Student's t test ;*p<0.05; n.s, not significant) .

Figure S4.

Morphological investigation of conditional TFR KO mice, Related to Figure 2

(A) Nissl staining on coronal section of WT and KO mouse brain at P20. **(B)** Neural cells of different brain regions (cortex, hippocampus, thalamus, hypothalamus) from WT and KO mouse. (20X objective) **(C)** Immunofluorescence co-staining of MAP2 (red) and SMI312 (green) on WT and KO mouse brain sections at P20. MAP2 and SMI312 are dendrite and axon markers, respectively. Scale bar = 200 μm.

Figure S5.

Deletion of TFR impairs synaptic number but not neuronal growth, Related to Figure 2

(A) Immunofluorescence staining of dendrite marker MAP2 on 7 and 12 *div* cultured hippocampal neurons. scale bar = 50 μm . Averaged total dendrite length of single neuron was measured for each group (n=20 neurons for each group, mean ± SEM, Student's t test ,*p<0.05; n.s, not significant). **(B)** Axon length of 7 *div* cultured hippocampal neurons. pEGFP plasmid was transfected to visualize axons as indicated by red arrows. scale bar = 50 μm. Averaged axon length was measured for WT and TFR KO group (n=20 neurons for each group, mean ± SEM, n.s, not significant). **(C)** Hippocampus and dendrite spines of WT and TFR KO Thy1-eGFP transgenic mice at P20 were outlined by green fluorescent proteins. large scale bar = 200 μm. small scale bar = 5 μm. **(D)** Quantification of dendrite spines density and average length (n= 60 dendrites from three pairs of WT and KO animals; mean ± SEM, Student's t test; *, p<0.05). **(E)** Immunofluorescence staining of synaptophysin and PSD95 on 14 *div* cultured hippocampal

neurons. scale bar = 20 μm.Positive puncta signal on dendrites were measured and quantified (n=10 neurons for each group; mean ± SEM, Student's t test; *, $p<0.05$).

Figure S6.

Total and surface protein expression levels of brain lysates and cultured neurons from WT and KO mice, Related to Figure 2 and Figure 6.

Total NMDAR1(NR1), GABA(B)R1 and AP2 protein samples were prepared as normal lyses method. Brain surface protein samples and neuron surface proteins samples were labeled with Sulfo-NHS-S-S-Biotin by biotinylation assay.

Figure S7.

AP2-GluR2 interaction in P2 fractions of mice brain, Related to Figure 6

Antibody against AP2 was used in the pull-down and then imunoblotted with GluR2 antibody. Two independent experiments were performed to confirm AP2-GluR2 interactions.

Movie S1

Epileptic seizure of TFR KO mouse, Related to Figure 2

Most of P20 TFR KO mice were attacked by seizure-like symptom, which was spontaneously developed or induced by external stimuli such as sharp sound. The progressive seizure usually undergoes several stages before coming of a sudden death: initiation stage, uttering intermittent squeaks and reduced movement; slight stage, begin to lose body WT with repeated muscle convulsion; moderate stage, whole body muscle spasm and facial convulsion; severe stage, intensive neural firing accompanied by faint.

Movie S2

Hindlimb clasp of TFR KO mouse, Related to Figure 2

P20 WT (left) and KO (right) mice were held upside down for 1min. Compared to WT animals, KO mice showed abnormal hindlimb clasp behavior. Also TFR KO mice were inclined to remain at rest with a sleepy eye.

Movie S3

Surface GluR1 and GluR2 trafficking during NMDA perfusion, Related to Figure 5

12*div* neurons of WT and KO were transfected with SEP-GluR1 or SEP-GluR2 constructs for 24hr before NMDA perfusion. Original fluorescent signal was recorded from FITC channel and visualized as black and white to maximize the image contrast. All neurons were imaged for 5 min before the stimulation with NMDA. The stimulation with 20 μM NMDA lasted for 5 min

following washout with normal perfusion buffer. The imaging process was recorded for 1hr and displayed as the accelerated timelapse video. The time stamp is minute, scale bar = 20 μm.

Extended Experimental Procedures

Primers

Genotype was determined by PCR with Nestin primer (5′-AGATGTTCGCGATTATC-3′ and 5′-AGCTACACCAGAGACGG-3′)and TFR (Forward 5’-CAGTAATCCCAGAGGAATCATTAG-3’ and Reverse 5’-CTAAACCGGGTGTATGACAATG-3’) primers. Reporter transgenic mice line contained a promoter-loxP-stop sequence-loxP-lacZ gene that can express bacterial β-galactosidase (β-gal) in the presence of Cre recombinase. The 5′ and 3′ primer for the Cre transgene (490 bp amplified) were 5′-AGATGTTCGCGATTATC-3′ and 5′-AGCTACACCAGAGACGG-3′, respectively. The 5′ and 3′ primer for the lacZ gene (825 bp amplified) were 5′-GACACCAGACCAACTGGTAATGG-3′ and 5′-GCATCGAGCTGGGTAATAAGCG-3′, respectively.

Antibodies

All antibodies used in the experiments were commercially bought, unless otherwise stated: PSD-95 (pAb, Cell Signaling Technology),TFR (mAb, Invitrogen),TF (mAb, Bethyl), AP2-A1

(mAb, Proteintech), GluR1-N (mAb, Millipore) and GluR1-C (mAb, Epitomics), GluR2-N (mAb, Millipore) and GluR2-C (mAb, Millipore), NR1 (mAb, Millipore), NR2A (pAb, Proteintech) and NR2B (mAb,NeuroMab).

Plasmids

The pJPA5-TFR-GFP construct was a gift from Gary Banker[1]. TFR sequence was amplified by PCR and inserted into the C-terminus of pcDNA3.1-zero-mCherry to generate TFR-mCherry. Results were sequenced by T7 promoter and verified by two enzyme digest, XbaI and EcoRI (New England Biolabs, Ipswich, MA). TFR-Y20A and TFR-F23A constructs were obtained by point mutation of TFR-mCherry at C-tail[2]. pH-sensitive constructs pCI-SEP-GluR1 (Addgene) and pCI-SEP-GluR2 (Addgene) were both commercially bought and were generated by Kopec group[3].

Histology

The cryosections (10 μm) of P20 mice brains were fixed in 4% paraformaldehyde in PBS buffer (pH =7.4) for 10 minutes, washed twice with PBS and then stained for X-Gal for up to 20 hr at room temperature in 1 mg/ml X-Gal (4-chloro-5-bromo-3-indolyl-β-galactosidase), 4 mM $K_4Fe(CN)_6$ 3H_2O, 4 mM $K_3Fe(CN)_6$, and 2 mM $MgCl_2$ in PBS. The slides were then washed with PBS twice, distilled water once and mounted in resin. Overall brain morphology was revealed by cresyl

violet (Nissl staining). Light microscopy was performed at 10x–63x magnification on a Nikon microscope.

Perl's stain for iron level

Briefly, paraffin sections were immersed in standard Perl's solution for 30 min and washed by ddH_2O. Add 0.3% H_2O_2 to block endogenous peroxidase. Then wash with PBS and add 3,3'-diaminobenzidine (DAB) at room temperature for 10 min. Stop reaction with PBS and seal sections with neutral resin.

Immunofluorescence staining

12-14 div neurons were fixed in 4% (w/v) paraformaldehyde and 4% sucrose in 0.01M PBS for 15 minutes and then washed three times in PBS each for 5 min. Cells were permeabilized with 1x PBS containing 0.25% (w/v) Triton X-100 for 15 minutes and blocked in blocking buffer (3% BSA, 2% FBS in 1XPBS) for 1hr at room temperature. Primary antibodies diluted in blocking buffer was added to incubate with cells overnight at 4 °C. Fluorophore-conjugated (Alexa-488, Alexa-555, Alexa-647) secondary antibodies were then added to bind primary antibodies. Coverslips were mounted with anti-fade medium and observed under 20x or 63x objectives.

Immunoprecipitation

The P2 fraction was lysed in buffer (20 mM Tris-HCl, 1 mM EDTA, 150 mM NaCl, 1% Triton X-100, protease inhibitor cocktail-EDTA [Thermo], pH=7.4) and then centrifuged at 10,000 g for 15 min at 4 ℃. The supernatant was incubated with antibodies overnight at 4 ℃ and then isolated with protein A/G-agarose beads (Santa Cruz).

Brain fraction

Subcellular fraction was performed as previously described, with minor modifications. Briefly, whole mouse cortex and hippocampus tissues were homogenized in ice-cold homogenization buffer (10 mM Tris-HCl, 5mM EDTA, 320mM sucrose, 1mM PMSF, PH 7.4). The homogenates were centrifuged at 800 g for 10 min to obtain nuclei and large debris (P1) and supernatant (S1). The S1 fraction was centrifuged at 10,000 g to obtain a crude synaptosomal fraction (P2) and supernatant (S2). The S2 fraction was centrifuged at 165,000 g to obtain a cytosol fraction (S3) and a light membrane/microsome enriched fraction (P3). The P2 fraction was lysed hypo-osmotically and centrifuged at 25,000 g to pellet a synaptosomal membrane fraction (LP1) and supernatant (LS1). The LS1 fraction was then centrifuged at 165,000 g to obtain the crude synaptic vesicle-enriched fraction (LP2) and supernatant (LS2).

AMPA Receptor Internalization Assay

After being cultured for 14 days, hippocampal neurons were labeled for 15 min at 37 ˚C with GluR1 or GluR2 N-terminus targeted antibodies (Millipore) in maintenance medium (10 µg/ml).

and then incubated in normal medium or medium with drugs (100 μM AMPA or 50 μM CNQX and 50 μM NMDA) for 10 minutes. After fixation for 15 min at room temperature in 4% paraformaldehyde/4% sucrose, neurons were stained with Alexa-488 conjugated secondary antibodies for 1 hr at room temperature without permeabilization to visualize pre-labeled surface receptors, and then permeabilized for 1 min in 100% methanol at -20 ℃ to label with Alexa-555 conjugated secondary antibodies and visualize pre-labeled internalized receptors.

Cell Surface Receptor BS^3-crosslinking Assay

To investigate surface distribution of AMPA receptors in TFR KO neurons in vivo, a BS3-crosslinking assay was performed as previously described [4,5]. Briefly, hippocampus tissues from TFR KO mice and WT littermates were rapidly removed and chopped into small pieces on ice and then crosslinked with 2mM BS3 (Pierce Biotechnology) in ice-cold PBS by 30 min incubation at 4 ℃ with gentle agitation. After the remaining BS3 being quenched by addition of 100 mM glycine (10 min at 4 ℃), the tissues were lysed in ice-cold RIPA-lyses buffer (50 mM Tris-HCl，150 mM NaCl，1% Triton X-100，1% sodium deoxycholate, 0.1% SDS, protease inhibitor cocktail-EDTA [Thermo], pH 7.4) for 1hr and then centrifuged at 10,000 g for 10 min. SDS-PAGE and western blot were performed to analyze the surface and intracellular pools of AMPA receptors.

Surface Biotinylation Assay

Cell surface receptor biotinylation assay was performed as previously described [6,7] with minor

modification. Briefly, high density neurons were cooled down to 4 ℃ and washed with ice-cold PBS. After being incubated with 1 mg/ml Sulfo-NHS-S-S-Biotin (Pierce) for 30 min at 4 ℃ on a shaker with gentle speed the remaining reactive biotin was quenched with Tris-buffered saline (50 mM Tris-HCl, pH7.4, 150 mM NaCl) and washed 2 times with ice-cold PBS before the cells were lysed in RIPA buffer and harvested by centrifugation at 10,000 g for 15 min at 4 ℃. One-fifths of each supernatant was denatured and used as a reference of total input. Four-fifths of each supernatant was incubated with 100 µl of 50% NeutraAvidin agarose beads (Pierce) for 2 hours at 4 ℃ on a rotator to isolate biotin-labeled proteins and then washed 3 times with RIPA buffer before heating at 100°C for 10 min in 100 µl of 2 X SDS loading buffer with 50 mM DTT.

Electrophysiology

Hippocampal slice preparation. Mice (P14-15) were anesthetized with halothane and decapitated. Hippocampi were rapidly removed and placed in ice-cold saline containing (in mM) 110 choline chloride, 2.5 KCl, 1.2 NaH2PO_4, 25 $NaHCO_3$, 0.5 $CaCl_2$, 7 $MgCl_2$, 2.4 pyruvate, 20 glucose, adjusted to pH 7.4 and oxygenated with 95% O_2/5% CO_2. Transverse slices (400 µm) were prepared with a vibrating tissue slicer (Leica VT1000S) and kept in ACSF containing (in mM) 126 NaCl, 5 KCl, 2 $MgCl_2$, 2 $CaCl_2$, 1.25 NaH_2PO_4, 26 $NaHCO_3$, 20 glucose, pH 7.4 for at least 1 hr at room temperature.

Field potential recording. Slices were transferred to a Haas-style interface recording chamber

(Warner Instrument) perfused with ACSF, warmed to 32.0±0.37℃ at a rate of 2 ml/min and allowed to recover for at least 1 hr. Field potentials were recorded with glass micropipettes (1-2 MΩ) filled with ACSF and placed in hippocampal CA1 stratum radiatum. Schaffer collateral/commissural fibers were stimulated at 0.05 Hz with brief (100 μs) constant-voltage pulses delivered by a concentric bipolar electrode (FHC). At the beginning of each experiment, an input/output curve was established and the strength of the test stimuli was adjusted to produce a response equal to ~50% of the maximum fEPSP slope. Signals were low-pass filtered at 2 kHz and digitized at 10 kHz. GABAzine (10 μM) was added to block $GABA_A$ receptors. To elicit LTP, two different stimulus protocols were used. In one set of experiments, theta-burst stimulation was used, consisting of five sets with an interest interval of 20 s. Each set consisted of 15 bursts delivered at 5 Hz, and each burst contained five pulses delivered at 100 Hz. In another set of experiments, weak titanic stimulation consisting of 50 pulses delivered at 50 Hz was used to evoke a smaller LTP response. All values were expressed as mean±SEM and compared statistically using unpaired Student's t-test. All chemicals were purchased from Sigma unless stated otherwise.

Supplementary References

1 Burack, M. A., Silverman, M. A. & Banker, G. The role of selective transport in neuronal protein sorting. *Neuron* **26**, 465-472, (2000).

2 Farias, G. G. *et al.* Signal-mediated, AP-1/clathrin-dependent sorting of transmembrane receptors to the somatodendritic domain of hippocampal neurons. *Neuron* **75**, 810-823, (2012).

3 Kopec, C. D., Li, B., Wei, W., Boehm, J. & Malinow, R. Glutamate receptor exocytosis and spine enlargement during chemically induced long-term potentiation. *J Neurosci* **26**, 2000-2009 (2006).

4 Conrad, K. L. *et al.* Formation of accumbens GluR2-lacking AMPA receptors mediates incubation of cocaine craving. *Nature* **454**, 118-121 (2008).

5 Zhang, J. *et al.* The AAA< sup>+</sup> ATPase Thorase Regulates AMPA Receptor-Dependent Synaptic Plasticity and Behavior. *Cell* **145**, 284-299 (2011).

6 Chung, H. J., Qian, X., Ehlers, M., Jan, Y. N. & Jan, L. Y. Neuronal activity regulates phosphorylation-dependent surface delivery of G protein-activated inwardly rectifying potassium channels. *Proc Natl Acad Sci* **106**, 629-634 (2009).

7 Mao, L., Takamiya, K., Thomas, G., Lin, D.-T. & Huganir, R. L. GRIP1 and 2 regulate activity-dependent AMPA receptor recycling via exocyst complex interactions. *Proc Natl Acad Sci* **107**, 19038-19043 (2010).

Figure S1

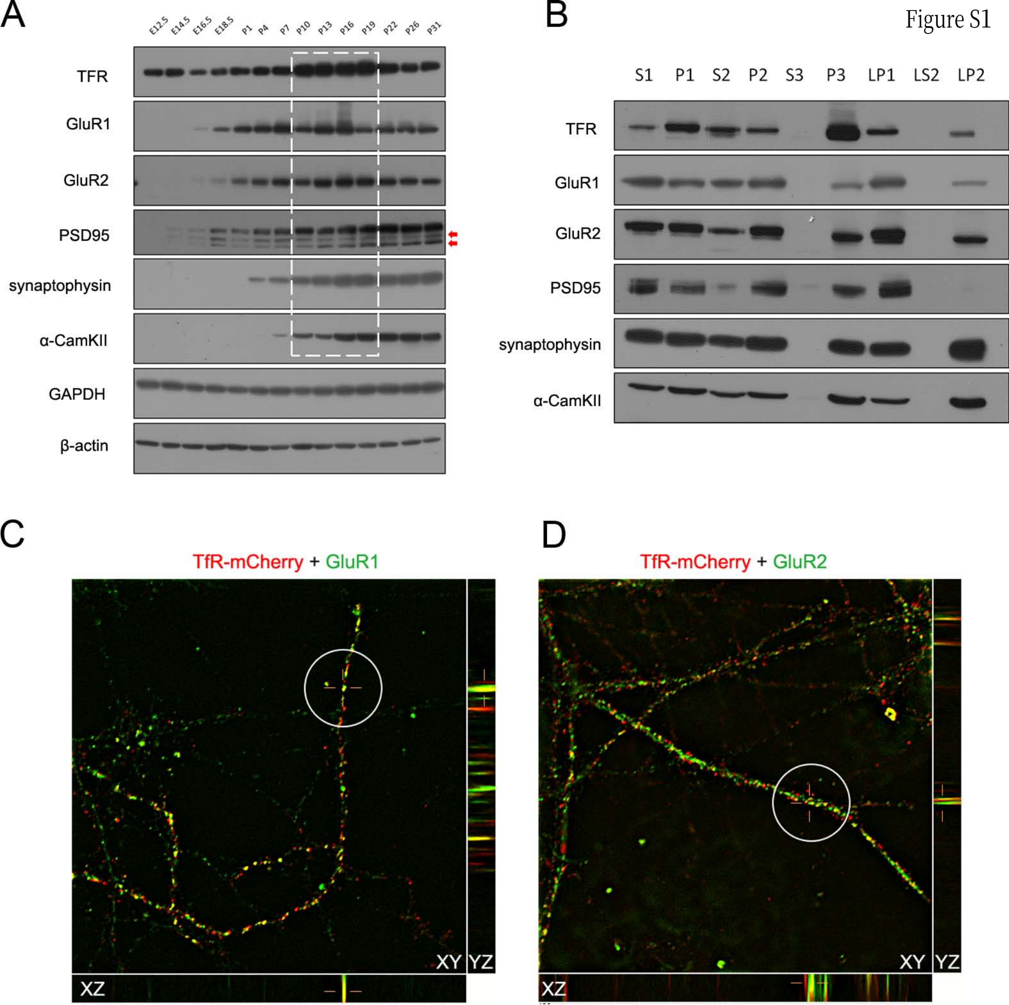

A
E12.5 E14.5 E16.5 E18.5 P1 P4 P7 P10 P13 P16 P19 P22 P26 P31
TFR
GluR1
GluR2
PSD95
synaptophysin
α-CamKII
GAPDH
β-actin
B
S1 P1 S2 P2 S3 P3 LP1 LS2 LP2
TFR
GluR1
GluR2
PSD95
synaptophysin
α-CamKII
C
TfR-mCherry + GluR1
XY
YZ
XZ
D
TfR-mCherry + GluR2
XY
YZ
XZ

Figure S2

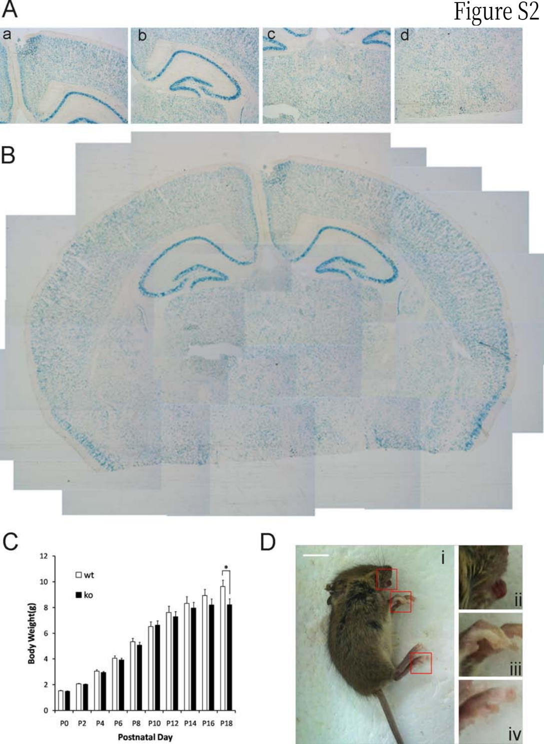
A
a
b
c
d
B
C
D
Body Weight(g)
12
10
8
6
4
2
0
wt
ko
*
P0
P2
P4
P6
P8
P10
P12
P14
P16
P18
Postnatal Day
i
ii
iii
iv

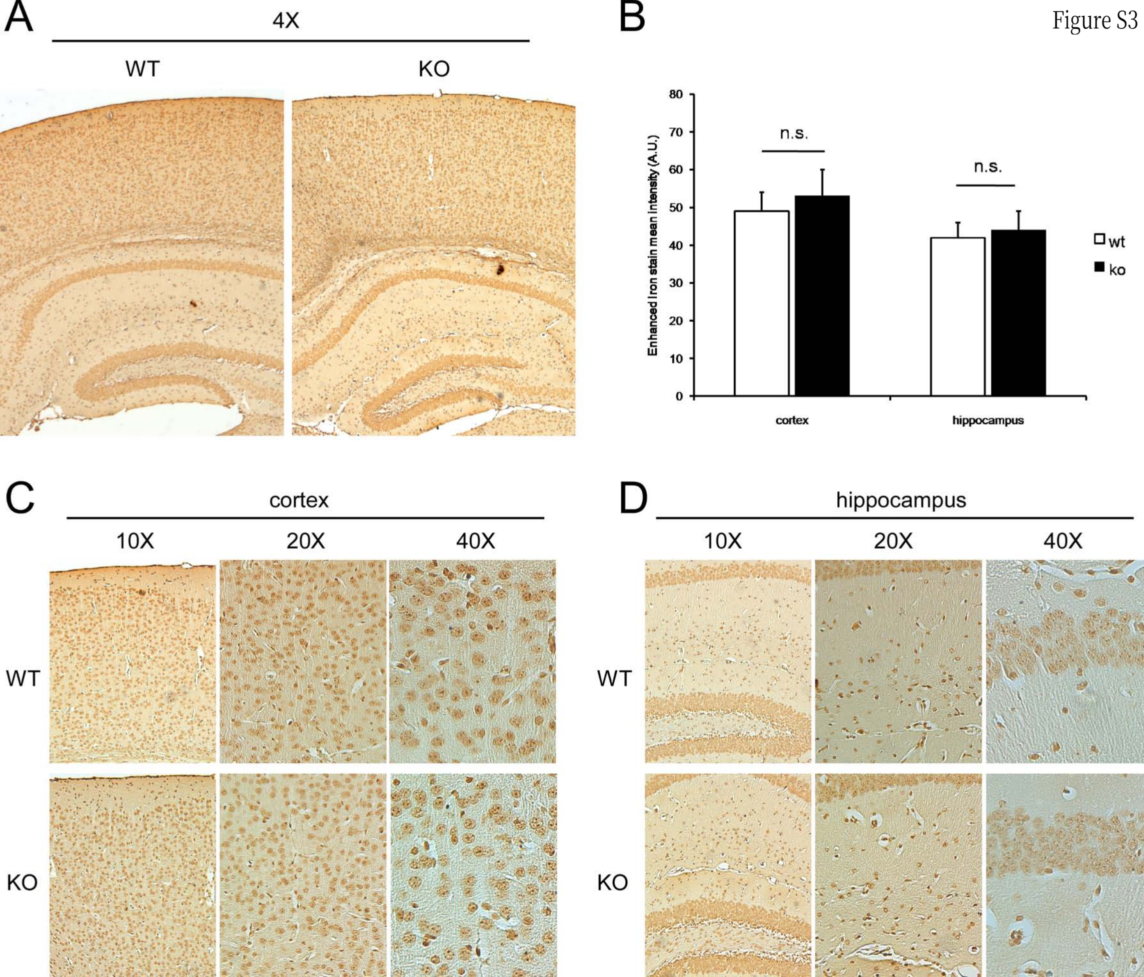
A
4X
WT
KO
B
Enhanced Iron stain mean intensity (A.U.)
80
70
60
50
40
30
20
10
0
n.s.
n.s.
wt
ko
cortex
hippocampus
C
cortex
10X
20X
40X
WT
KO
D
hippocampus
10X
20X
40X
WT
KO

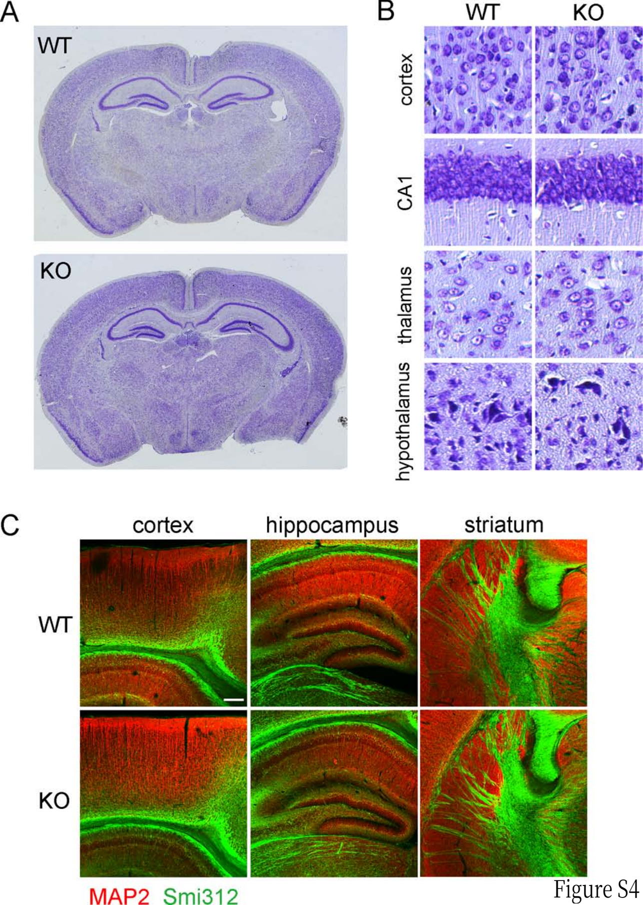

A
WT
KO
B
WT
KO
cortex
CA1
thalamus
hypothalamus
C
cortex
hippocampus
striatum
WT
KO
MAP2 Smi312

Figure S4

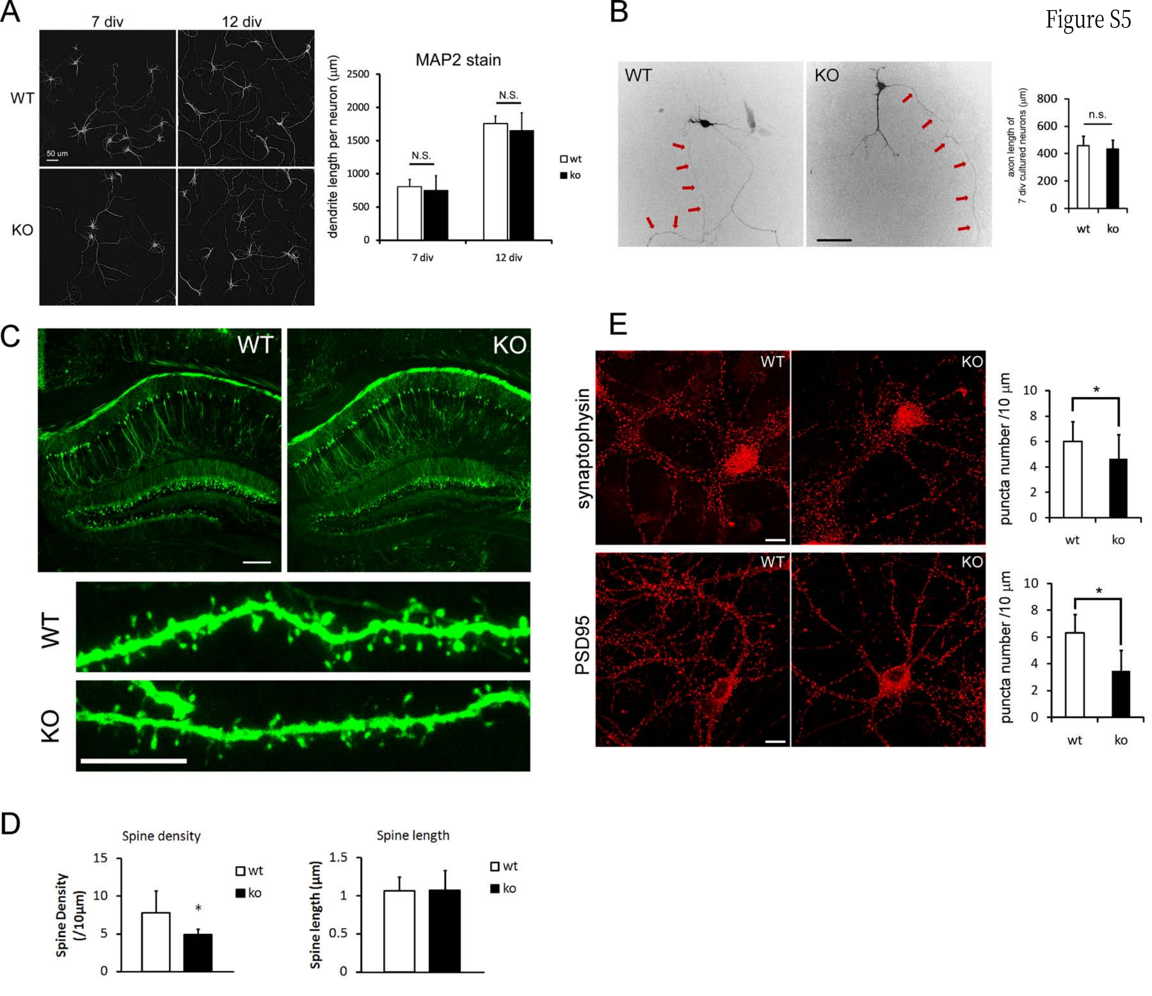

Figure S5
A
7 div
12 div
WT
KO
50 um
MAP2 stain
dendrite length per neuron (µm)
N.S.
N.S.
wt
ko
7 div
12 div
B
WT
KO
axon length of 7 div cultured neurons (µm)
n.s.
wt
ko
C
WT
KO
WT
KO
D
Spine density
Spine Density (/10µm)
wt
ko
*
Spine length
Spine length (µm)
wt
ko
E
synaptophysin
WT
KO
puncta number /10 µm
*
wt
ko
PSD95
WT
KO
puncta number /10 µm
*
wt
ko

Figure S6

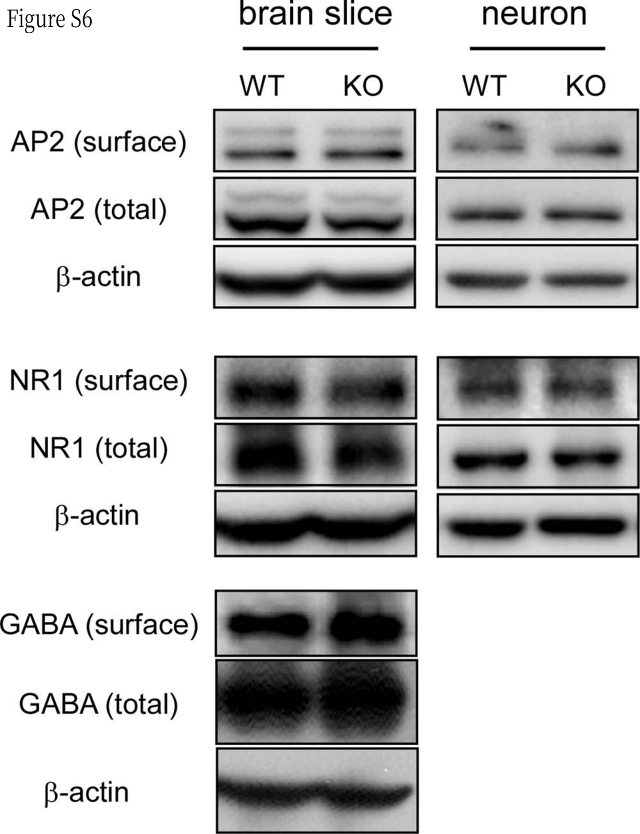
brain slice
neuron
WT
KO
WT
KO
AP2 (surface)
AP2 (total)
β-actin
NR1 (surface)
NR1 (total)
β-actin
GABA (surface)
GABA (total)
β-actin

Figure S7

Experiment 1

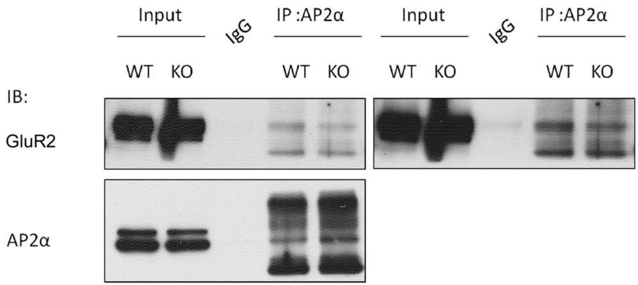

Experiment 2

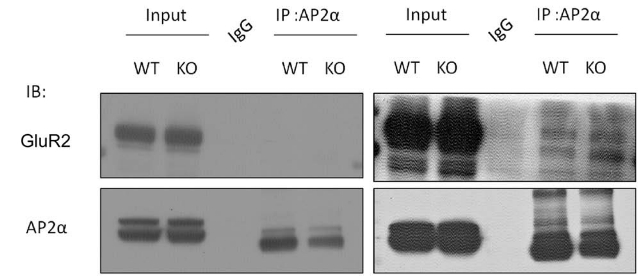

Supplement: Supplementary Information [file srep21019-s1.pdf]
